# A Severity Comparison between Italian and Israeli Rett Syndrome Cohorts

**DOI:** 10.3390/diagnostics13213390

**Published:** 2023-11-06

**Authors:** Alberto Romano, Meir Lotan, Rosa Angela Fabio

**Affiliations:** 1 Department of Health System Management, Ariel University, Ariel 4070000, Israel; 2 Department of Physiotherapy, Ariel University, Ariel 4070000, Israel; 3 Israeli Rett Syndrome National Evaluation Team, Ramat Gan 5200100, Israel; 4 Department of Economics, University of Messina, 98122 Messina, Italy

**Keywords:** Rett syndrome, cross-cultural comparison, Rett assessment rating scale, severity of illness index, mainstreaming education, special education, inclusive education, developmental disability

## Abstract

Rett syndrome (RTT) is a neurodevelopmental disorder marked by profound cognitive, communication, and motor impairments. Despite identified genotype/phenotype connections, the extent of clinical severity varies even among individuals sharing the same genetic mutation. Diverse sociocultural environments, such as the level of inclusivity of the scholar system, the time spent with family, and the intensity of the rehabilitative intervention provided, might influence their development diversely. This study examines the severity of RTT in people in Italy and Israel, countries with distinct contradictory approaches to caring for those with intricate disabilities, across two age groups. Data from 136 Italian and 59 Israeli girls and women with RTT were assessed and divided into two age categories: above and below 12 years. The RARS, a standardized RTT-specific clinical severity tool, was administered. Despite no differences in age and genetic characteristics, the Italian group showed better scores in the RARS motor and disease-related characteristics areas in both age groups. Moreover, the young Italian participants gathered better total RARS scores and emotional and behavioral characteristics area scores. Furthermore, the young group showed significantly less scoliosis, foot problems, and epilepsy than the older group. These findings endorse the inclusion of girls with RTT in the regular schooling system for a limited daily period, investing in high activity levels within the home and community environments, and suggest continuously providing the person with daily occasions of physical activity and socialization.

## 1. Introduction

Rett syndrome (RTT) is a neurodevelopmental syndrome that affects about 7.1 per 100,000 females worldwide [1], making it the second most common multi-disability syndrome in females after Down syndrome [2]. RTT is a complex disorder that impacts various physical and neurological development aspects. A hallmark feature of RTT is the regression of functional communication and motor skills, occurring after a period of normal development following birth.

Cognitive abilities in individuals with RTT show wide variations [3], with cognitive assessment complicated by the communicative and physical limitations experienced by these people [4,5,6,7]. Verbal communication in people with RTT is typically absent or limited to a few words or phrases [8,9,10,11] due to difficulties in movement planning and coordination [6,8,9]. However, many individuals with RTT have learned to use augmentative and alternative communication (AAC) methods with intent [10,11,12]. The most commonly reported communication modalities involve eye gaze, body movements, and electronic and non-electronic AAC systems, such as communication boards with symbols and pictures [10,11,13].

After the regression phase, patients with RTT experience challenges in coordination and balance due to uncontrolled movements of body segments and trunk, which affect their gross motor function [14,15,16,17,18]. Although most patients retain residual gross motor abilities until adulthood, around half can walk independently or with minimal support. However, from age 13 onwards, a decline in motor function quality becomes evident, leading to an increasing reliance on support as they age [19]. Moreover, fluctuations in muscle tone and the emergence of compensatory muscle rigidity have been observed from early childhood [20]. RTT also presents neuromuscular impairment and musculoskeletal abnormalities, with prevalent deformities affecting the spine and feet, although all body joints may be affected [21].

Additionally, behavioral functioning can be affected, with episodes of social withdrawal, mood swings, anxiety, and mostly self-directed aggressiveness [22,23,24,25]. Physiological alterations and dysfunctions, such as seizures, abnormal breathing and sleeping patterns, and growth retardation in the head and extremities, have also been reported [26,27]. The severity of these aspects of RTT varies from mild to severe manifestation, and their combination and interaction determine the severity of the disorder for each individual. Although genotype/phenotype relationships have been reported, linking disease severity to specific genetic mutations [28,29], clinical severity remains variable even among individuals with the same mutation, limiting the prognostic value of these relationships [30].

### 1.1. Comparing Israeli and Italian Patients with RTT

Although genetics plays a significant role in the severity of RTT, environmental factors also influence the disorder’s developmental trajectory, particularly regarding functional abilities. Recent literature suggests that daily activities can enhance cognitive [31,32], communicative [33,34], and motor skills [35,36,37] in individuals with RTT. These findings indicate that an active, participative, motivational, and demanding environment can promote the development of functional abilities in people with RTT. Therefore, different sociocultural environments could have varying impacts on the development of these individuals.

Conducting a comparison between girls and women with RTT from Israel and Italy can be highly valuable in the field of scientific research for several reasons:

- Genetic and environmental factors—Comparing individuals from different geographical regions allows researchers to investigate potential variations in genetic backgrounds and environmental factors that may influence the presentation and progression of RTT. Differences in genetic mutations or environmental exposures could contribute to distinct clinical features or responses to treatments.
- Phenotypic variability—RTT is known for its phenotypic variability, meaning that individuals with the same genetic mutation can exhibit a wide range of symptoms and functional abilities. By comparing individuals from different populations, researchers may identify specific patterns of symptoms and variations in the syndrome’s clinical manifestations.
- Therapeutic approaches—Comparative studies can shed light on the effectiveness of the different therapeutic approaches used in Israel and Italy. Variations in medical practices, therapeutic interventions, and healthcare systems could influence outcomes and provide valuable insights into optimizing treatments for RTT.
- Data generalizability—Research findings based on a diverse sample of individuals from Israel and Italy can lead to more generalizable conclusions about RTT. Having data from multiple populations strengthens the external validity of research studies, allowing for a broader application of findings to other populations worldwide.
- Cross-cultural perspectives—Comparing RTT cases between countries enables a cross-cultural perspective on caregiving practices, societal support, and family dynamics. Such an approach can help identify cultural factors that may influence the quality of life and care received by individuals with RTT.
- Identifying best practices—If there are notable differences in treatment outcomes or management strategies between the two countries, a comparative analysis can help identify “best practices” that lead to improved quality of life and functional outcomes for individuals with RTT.

### 1.2. Models of Caring in Italy and Israel

Italy and Israel represent examples of countries that have adopted different models of caring for people with complex disorders, including those with RTT, each with its pros and cons.

In Italy, children and adolescents with complex disabilities participate in mainstream schools with their typically developed peers, sometimes until adulthood, for an average of six hours a day (from 8 a.m. to 2 p.m.), five days a week (total = 30 h a week), within an inclusive model of schooling. This inclusion into mainstream schools allows for daily interaction with typically developed individuals, which can activate attention and communication through participation. Each girl with RTT in Italy is supported by a dedicated team consisting of a special education teacher and an educator with expertise in the field. This multidisciplinary team collaborates closely to ensure educational activities are tailored to each girl’s needs, allowing participation with the typically developed classmates. The special education teacher plays a pivotal role in preparing educational materials aligned with the functional assessment of the girls. However, a drawback is that mainstream schools may struggle to offer specialized and knowledgeable assistance to individuals with highly complex conditions. After school, usually after lunch, these individuals stay at home with their families and may be enrolled in rehabilitation or educational activities in specialized facilities. However, as individuals with disabilities age into adulthood, the services provided by the system often reduce, and adults with complex disabilities may either stay at home with their families or attend daily centers with other adults with disabilities. Consequently, during adulthood in Italy, they receive few or no rehabilitative interventions from the system.

On the other hand, in Israel, people with complex disabilities attend special schools or centers with other individuals with disabilities across all age groups and for the entire day (usually nine hours a day, from 8 a.m. to 5 p.m., five days a week, plus five hours a day, from 8 a.m. to 1 p.m. one day a week—total = 50 h a week). In these schools, they receive specialized assistance and rehabilitation treatment throughout their lives. Although this model provides better assistance and dedicated care and allows both parents to have full-time jobs, participation opportunities with typically developed peers are limited, and interactions are frequently restricted to the educational staff. Nevertheless, the Israeli system provides families of people with complex disabilities with a caregiver dedicated to the individual, supporting the family’s daily living and well-being.

### 1.3. Study Objectives

The current study aims to evaluate the severity level of girls and women with RTT living in Italy and Israel, two countries with different approaches to caring for people with complex disabilities.

The second objective is to analyze the severity levels at different age levels, as this factor may provide important insights into the progression and management of RTT over time. By comparing the severity levels between the two countries and across different age groups, the study seeks to identify potential differences and patterns that can contribute to a better understanding of the impact of environmental and cultural factors on the development and management of RTT.

In conclusion, this comparative study between Israeli and Italian patients with RTT holds significant potential for advancing scientific knowledge about RTT, its clinical presentation, and the efficacy of different care models. By considering both genetic and environmental influences, researchers can gain valuable insights into the complexities of the disorder, leading to improved interventions and support for individuals with RTT and their families.

## 2. Materials and Methods

### 2.1. Participants

The current retrospective preliminary observational study enrolled 136 Italian (mean age 14.5 ± 7.9 years) and 59 age-matched Israeli (mean age 14.4 ± 7.9 years) girls and women with RTT (Tot = 195). Participants’ data were retrieved from the databases of the Italian and Israeli Rett Syndrome Associations. To enroll in this study, the participants had to be diagnosed with RTT in its classic form [15], aged between 3 and 40 years, reside at home with their families, and have lived their lives in Italy or Israel. Moreover, their legal kin must have provided informed consent for anonymized data use for research purposes.

Participants were divided into two age groups: between 3 and 11 years (U11 group) and between 12 and 40 years (U40 group), as done in a previous study on aging in RTT [38]. The Italian U11 group included 55 individuals (mean age 7.2 ± 2.2 years) and the U40 group 81 (mean age 19.6 ± 6.2 years), while the Israeli age groups included 21 (mean age 6.2 ± 2.3 years) and 38 (mean age 18.9 ± 6.0 years) participants, respectively.

Specific details of MeCP2 gene mutations were available for 106 (54.3%) participants. The specific MeCP2 mutations identified in the Italian and Israeli groups are presented in Table 1.

Specific information about the intensity of ongoing rehabilitation interventions at the time of the data collection was available for 166 participants (85.1%). The intensity of the rehabilitation treatment was divided into three categories based on the number of received treatments: none or not intensive (between 0 and 1 treatment per week), semi-intensive (between 2 and 4 treatments per week), and intensive (more than 4 treatments per week).

### 2.2. Rett Assessment Rating Scale

Rett Assessment Rating Scale (RARS)—The RARS is an RTT-specific clinical severity tool implemented in several research studies [40,41]. It is a standardized tool developed to assess and monitor symptoms’ clinical severity and progression in girls diagnosed with RTT [42]. The RARS consists of items that evaluate various domains of functioning in individuals with RTT, including physical abilities, motor skills, communication, and social behavior. The RARS allows for a comprehensive and accurate evaluation of girls with RTT by individually analyzing and assessing specific characteristics. Out of the 31 items comprising the RARS, 30 have been grouped into six areas of assessment:

- Cognitive area—This area addresses the compromised cognitive abilities in girls with RTT. Due to the initial regression, their cognitive level typically remains severely delayed. Precise indicators related to cognitive development include attentional abilities, spatial and temporal orientation, memory, verbal communication skills, non-verbal communication through facial expressions, the ability to maintain eye contact and shared attention, and the presence of responsive smiling.
- Sensory area—Girls with RTT may experience visual issues characterized by peripheral gaze and hearing problems involving fluctuations in auditory sensitivity. Therefore, two items related to vision and hearing are included in the RARS.
- Motor area—Motor difficulties in girls with RTT primarily affect their walking ability and hand stereotypes. The diagnostic criteria for RTT include the appearance of hand stereotypies such as hand-washing, hand-clapping, and hand-wringing, as well as the emergence of ataxic and apraxic gait and trunk movements. The motor area of the RARS includes four items related to the body, hands, scoliosis, and feet.
- Emotional area—People interacting with girls with RTT find it easy to establish connections with them as they respond to social stimuli and smile with an intense gaze. Their emotional states are typically related to their well-being [42]. The items in the emotional area concern the basic emotions (assessing the ability to express and understand emotions, including the emotions of others), mood swings, and anxiety, which are common in individuals affected by the syndrome.
- Autonomy area—This area includes evaluating the control of sphincters, the ability to feed independently, and skills related to personal hygiene, such as washing and dressing.
- Typical characteristics of RTT—These can be categorized into disease-related and behavioral features. The items related to disease-related features investigate the presence and intensity of epilepsy, convulsions, dyspnea crises, and aerophagia. The items concerning the behavioral features explore the presence of hyperactivity, aggressiveness, bruxism, eating preferences, oculogyric crises, and muscle tension.
- The last item of the test (no. 31) refers to the overall impression that parents or therapists, who fill out the RARS, have regarding the severity of the disease in the child.

The scale is typically administered through structured observations and caregiver reports, ensuring a multi-dimensional evaluation of the person’s abilities and difficulties. The items are rated based on the frequency and severity of observed behaviors and symptoms. The individual’s characteristic is rated on a 7-point discrete scale as follows: 1 = task completion is independent/within the normal limits/absence of deficit; 2 = task completion is independent but with difficulties or only sporadically/low deficit level; 3 = task completion requires support/moderate to severe deficit; 4 = task completion is not possible/severe deficit. Intermediate values (1.5, 2.5, and 3.5) are used when the answer does not precisely match any integer value. Therefore, lower values represent a better outcome for the rated person. Consequently, the RARS quantifies symptom severity, disease progression, and intervention response over time. The Italian version of RARS was validated with 220 RTT patients, confirming its statistical validity and reliability. Score analysis showed a normal distribution with mean scores similar to median and mode. Skewness and kurtosis values for total score distribution were 0.110 and 0.352, indicating normality. Cronbach’s alpha showed high internal consistency for the total scale (0.912) and subscales (0.811–0.934) [28].

Utilizing the RARS as an assessment tool can yield several valuable benefits. First, it facilitates early diagnosis and intervention by detecting the presence and severity of RTT-related symptoms. Early intervention is crucial to optimize treatment outcomes and support the individual’s development. Second, the scale allows for the monitoring of disease progression, enabling healthcare professionals to tailor therapies and interventions according to the person’s changing needs. Moreover, the standardized nature of the RARS enhances the comparability of data across different patients and research studies, contributing to a more comprehensive understanding of the syndrome and the effectiveness of therapeutic approaches.

The RARS was administered during the routine evaluations conducted by the Italian Rett Syndrome Association’s rehabilitation professionals and the Israeli Rett syndrome national evaluation team between 2016 and 2018. Both teams comprise rehabilitation personnel experienced in evaluating and treating people with RTT. The participants’ primary caregivers filled in the scale with the supervision of the rehabilitation personnel of the national evaluation teams, who remained available until the end of the scale administration to provide clarifications and help if needed.

### 2.3. Data Analysis

The ages and the intensity of ongoing rehabilitation treatments of participants in the Italian and Israeli age groups were compared using the Mann–Whitney’s U test. The same test compared the RARS items, areas, and total score distributions across age groups. The MeCP2 mutation distributions in the Italian and Israeli groups were compared through the chi-squared statistic. MeCP2 mutations were grouped into three main categories in accordance with a previous protocol [39]. The first pertains to mutations that affect the N-terminal domain (NTD) that modulate the ability of MeCP2 to interact with DNA [43] as well as influence the turnover rate of the protein [43,44]. The second category affects the methyl binding domain (MBD), constituting the sole structurally organized segment of MeCP2. This segment affects the tertiary structure (folding) [45] of this region, thus influencing its binding affinity [46]. The third category includes mutations affecting the rest of the molecule. A significant number of the remaining mutations are situated in the C-terminal domain CTD [47,48] and can affect the interactions of MeCP2 with many of its diverse interaction partners [49], including the chromatin itself [50] and RNA. Therefore, the distributions of the MeCP2 mutations within these categories (Table 1) were compared to assess the groups’ comparability. The threshold for statistical significance of the analysis mentioned above was set at α < 0.05.

## 3. Results

At the moment of data collection, 132 (97.1%) of the included Italian participants attend mainstreaming schools, and 58 (98.3%) of the Israeli girls attend special schools. No differences between the ages of participants in the Italian and Israeli age groups were found (Italian U11 vs. Israeli U11: *p* = 0.144; Italian U40 vs. Israeli U40: *p* = 0.601). Moreover, the available affected MeCP2 domain and intensity of rehabilitation treatment distributions do not differ between the two national groups (*p* = 0.096 and *p* = 0.231, respectively), supporting the comparability of the datasets.

**Figure 1:**
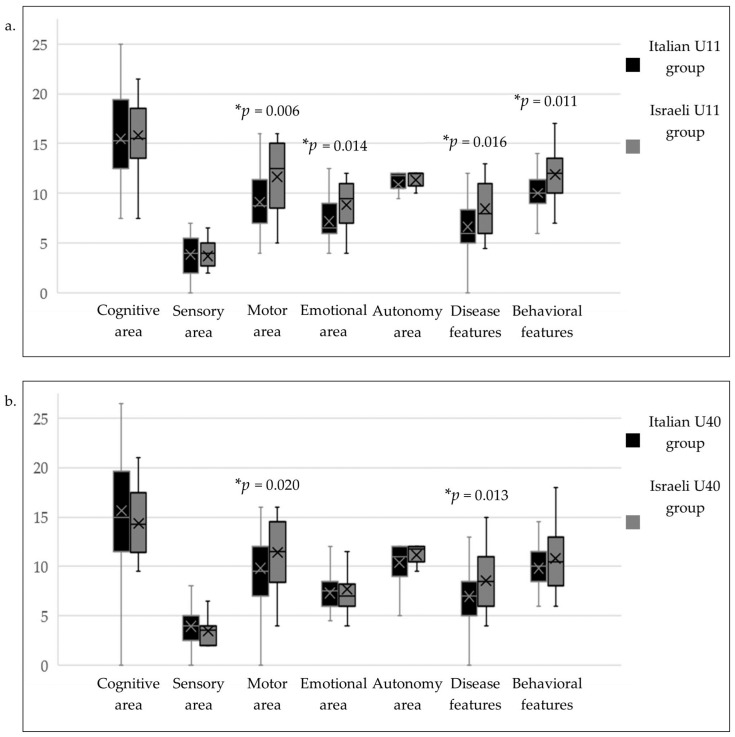
Graphical representation of the comparison between the Italian and Israeli participants’ RARS areas scores for the U11 (**a**) and U40 (**b**) age groups. The box inferior limits represent the 1st quartile of the distribution, and the upper limits represent the 3rd quartile (median excluded). The lines across the box show the median score of each group. The crosses inside the boxes mark the mean value of each dataset. The whiskers indicate the minimum and maximum distribution values (outliers identified through Tukey’s method excluded). *: *p* < 0.05.

The results of the RARS scores comparison between the Italian and Israeli age groups are reported in Table 2. Individual age and RARS scores of all participants, the relative descriptive statistics, and the statistical comparison results are available as Supplementary Materials (Tables S1–S3). On average, the Italian group obtained lower RARS scores in both age groups’ motor areas (U11 *p* = 0.006; U40 *p* = 0.020). The same difference was found for the disease-related characteristics (U11 *p* = 0.016; U40 *p* = 0.013). Moreover, the young Italian participants (U11 group) gathered better (lower) scores in the emotional area (*p* = 0.014), behavioral characteristics (*p* = 0.011), and total RARS score (*p* = 0.039). However, these differences flatten in the older (U40) group. The comparisons between the RARS area scores of the age groups are depicted in Figure 1.

To analyze the nature of the between-group differences in the RARS areas more deeply, the individual RARS items were analyzed. Within the Emotional area, the U11 Italian group scored better in the “basic emotion” item (*p* = 0.017), showing a greater ability to express their emotion to others. This statistical difference was not found between the U40 groups. Regarding the Motor area items, the U11 Italian participants showed less severe scoliosis (*p* < 0.001), but this difference was not found between the U40 groups. On the other hand, the U40 Italian group achieved better scores in the “body” (*p* = 0.026) and “hand” (*p* = 0.033) items, representing better walking and standing ability and more functional hand use than their Israeli peers. Looking at the items describing the disease-related features, Italian participants showed less convulsion and epilepsy than the Israeli group in both U11 (*p* < 0.001 and *p* = 0.033, respectively) and U40 (*p* < 0.001 and *p* = 0.006, respectively) age groups. Finally, between the items describing the behavioral characteristics of RTT, participants in the U11 Italian group showed less oculogyric crisis (*p* = 0.032) and eating preferences (*p* = 0.008) but presented higher hyperactivity behavior compared with the Israeli group (*p* = 0.007). These differences were maintained between the U40 groups (*p* = 0.013, *p* = 0.002, and *p* = 0.010, respectively) even though the behavioral features area did not differ between the two U40 groups (*p* = 0.236).

Although no statistically significant difference was found comparing the RARS areas and total scores between participants in the U11 and U40 age groups, analyzing the individual RARS items, differences between the age groups emerged. The U11 group showed significantly less scoliosis (*p* < 0.001), foot problems (*p* = 0.031), and epilepsy (*p* = 0.027) than the older participants. On the other hand, the U40 participants showed less bruxism (*p* = 0.027) and were more independent in feeding themselves (*p* = 0.050) than the U11 group. However, these differences were not found in the Israeli age groups. The U40 Israeli participants presented a better understanding of the others’ emotions (*p* = 0.029). No other significant differences were found.

## 4. Discussion

This preliminary study analyzed and compared the severity level of people with RTT living in Italy and Israel across two age groups. The findings generally suggest better significant scores of the Italian cohort in the “body”, “emotional”, “disease features”, and “behavioral features.” These differences decline with age and decrease when comparing the older participants of the two cohorts.

The two countries adopt different educational and support systems. The Italian educational system for people with complex disorders such as RTT follows an inclusive approach, allowing increased interactions with typically developed peers. Special educational schools are preferred in Israel as more professional care can be provided during the day, and both parents can attain full-time jobs. Therefore, in Italy, the girls return from school around noon, are kept active, and attain therapeutic interventions and higher activity levels within the house and the community than the Israeli children. On the other hand, Israeli children with RTT return from school at later hours (5–6 p.m.), and they are too tired to be involved in most types of activity. Therefore, Italian children with RTT are mostly more active during the day. An active lifestyle was suggested for many years by experts in the field of RTT [51,52] and was associated with a reduction of constipation [53], improved prognosis of scoliosis [35], reduced osteoporosis [52], and regulation of the autonomic system [54].

### 4.1. National Groups Comparison

Since both groups were not found to differ in age, genetic mutations, and intensity of ongoing rehabilitation intervention, most differences between the two national U11 groups may be explained by looking at the benefits of an inclusive education approach and a more active lifestyle. Daily practice of motor activities in an enriched environment providing social (e.g., engagement with other children), sensory (e.g., colorful room and songs), and cognitive (e.g., eye contact, age-appropriate activity with purpose, and praise for efforts and achievements) enhanced stimulations resulted in improved motor abilities of girls with RTT [54]. Most of these environmental enrichments are found in Italian educational settings, particularly within kindergartens and elementary schools, where children with disabilities are enrolled in the same motor activity as their peers (with the necessary facilitations to achieve success). During these activities, the class praises and recognizes the child’s effort and success, providing strong positive reinforcement to practice [55]. Due to the social nature of individuals with RTT [53], these types of encounters with neurotypical children are highly motivational for the child with RTT. It is important to emphasize that, given the nature of RTT, the girls do not follow the standard class curriculum. Instead, the focus is on fostering their involvement within the classroom group and promoting social inclusion. Therefore, interactions with peers are organized in a manner that respects the girls’ individual capabilities and needs. Throughout the school day, individualized programs are implemented to maximize the girls’ engagement and promote their development. These programs encompass moments of interaction with classmates, adapting educational activities to facilitate active participation, and the management of breaks during the day. The individualized facilitator of interaction, educational objectives, and strategies to enhance participation are discussed and identified in a multidisciplinary equipe composed of the class and special education teachers, the educator, and the referral clinicians (therapists and physicians). Although the educational day dynamics of each girl with RTT in Italy are peculiar and individualized, the general day schedule can be summarized as follows:

- Arrival at school—The day begins with the girl’s arrival and a meeting between the caregiver and the special education teacher to exchange information about the girl’s status.
- Morning roll call—During the morning roll call, the girl is addressed and responds to her name in the best way she can. This response may involve eye-tracking technology, raising a hand, or occasionally using her voice.
- Girls’ attendance and responses—This step marks the attendance and engagement of the girl in the class. For example, if the lesson regards food, the child must choose the food she prefers. The choice could be made adequately for the girl’s ability (e.g., using eye-tracking technology with two stimuli, raising a hand, or using her voice).
- Individualized programs—Individualized programs are implemented throughout the day to cater to the girl’s specific needs and abilities. For example, if the girl can discriminate between the photo of the mother and father, another photo is presented to discriminate between other people. If she can bring food to the mouth with help, the teacher tries to fade the help.
- Meeting moments with classmates—The girl has scheduled moments for interaction with her classmates. These interactions are structured to promote social engagement.
- Adaptation of educational activities for participation—Educational activities are adapted and modified to ensure active participation by the girl, considering her unique requirements.
- Snack time—A designated time for snack breaks is provided during the school day together with their peers to ensure that the girls’ nutritional needs are met, taking advantage of the sociality of the meal.
- End-of-Day Greeting—The school day concludes with an end-of-day greeting, providing closure to the day’s activities.

This structured approach allows the girl to be an integral part of the classroom environment and fosters her social inclusion and development.

Moreover, as the educational day ends at noon, the child has more time to participate in an active environment, walking, playing, and engaging in different activities and surroundings. On the other hand, children with complex disabilities participating in special educational programs practice their motor skills primarily within a rehabilitative setting, which allows for some effort and training but lacks the environmental enrichments mentioned above. These experiences within the community setting are extremely important, specifically for individuals diagnosed with apraxia [53], such as those with RTT, as they enable the generalization of specific skills practiced within the rehabilitative environment. Moreover, preliminary results support the benefits of adjunctive and active daily physical activity programs in preventing scoliosis progression in people with RTT [35]. Although Italian children with RTT do not routinely receive such programs, the enriched practice at regular schools and the activities that follow within the home setting after returning from school might promote better trunk muscular symmetry, reducing the entity of scoliosis in this group.

Conversely, Israeli children stay in the educational setting (mainly in a sitting position) throughout the day and return home too late to be engaged in different activities. This approach enables both parents to keep a full-time job but impose a sedentary lifestyle on the person with RTT, which results in a poorer prognosis exhibited by the scores achieved by the RARS. In line with these results, an analysis of several intervention programs summarized their findings by suggesting that an individualized physical program should be regularly recommended and implemented with individuals with RTT to preserve autonomy and improve their functional condition, therefore improving the quality of life [56].

It is of interest that the Italian U40 group showed better walking and standing ability and more functional hand use than their Israeli peers. As the Israeli system provides more support (i.e., dedicated caregivers/therapists), it was expected that older Israeli participants would experience more gross and fine motor practice. The authors believe this result could be due to a paradox: as Italian adolescents and adults spend their afternoons with their family members, they are prompted to walk around with them and stimulated to use their hands in their daily activities. On the other hand, Israeli participants stay at special education schools or facilities all day, practicing their functional motor abilities primarily within rehabilitation sessions, which are limited in time. In this context, the dedicated personnel take care of the daily needs of the person for her, eventually limiting the opportunity to participate in such activities, resulting in less practice of, for instance, hand use. These findings reiterate the importance of an active lifestyle for individuals with RTT, which is in line with the existing literature [57,58,59]

Moreover, pupils with multiple disabilities placed in less restrictive educational environments (mainstreaming education) showed increased social interactions, higher levels of social support from others, and more extensive friendship networks of peers without disabilities [60,61]. The higher number of occasions for social interactions allows for practice and improves the ability to express emotions and be understood by others, eventually explaining the higher RARS emotional score of the young Italian group. The lack of such a difference between the older national groups might be explained by the reduced opportunity for socialization for the Italian people when they graduate from school, which results in fewer daily experiences of their emotions being understood by others outside their family.

The increased opportunity for social interaction could also positively affect the number of oculogyric behaviors that are self-stimulative and social-seeking. The natural occurrence of social contact in general education classrooms may prevent this behavior from establishing, leading to its reduced presence in the older Italian group. Moreover, sharing mealtimes at school with peers or at home with the whole family allows feeding to be a positive experience and a joyful situation, favoring the prevention of food selectivity from establishing. However, the Italian children showed more hyperactive behaviors. The hyperactivity can be explained by the Italian group’s higher motor function level (making them appear more mobile, active, and movement-oriented) and by the activation caused by social interactions.

Finally, the Italian group shows lower (better) scores in the disease features RARS area, presenting with less epilepsy and convulsions than the Israeli participants in both age groups.

Considering the reflections mentioned above, the Italian participants with RTT seem to have benefited from the less restrictive educational environment with benefits for their social and motor development. The increased opportunity to interact with typically developed peers may have promoted positive and meaningful practice and efforts, resulting in better postural hygiene and emotional communication skills. It should be emphasized that to achieve such augmented educational environments, mere integration (just being there) is not enough; an inclusive environment is required [62]. Achieving effective scholar inclusion for people with complex disabilities is challenging. It requires specific resource allocation, effective protocols, and dedicated observations and planning. Below, the way these four aspects are managed in Italy is summarized.

- Resource allocation—It comprises the resources needed for dedicated personnel (special educational teacher, educator, support staff), structural interventions to overcome the architectural barriers (to make the scholar environment accessible), specific educational materials (including the time to construct it), and training courses (in general about inclusion strategies and specifically about the person’s disorder).
- Protocols—This refers to pre-established practices of the school as a system aimed at facilitating the inclusion process. It includes dedicated times for discussion (between the teachers, family members, and clinicians) and training courses, specific evaluation protocols of the inclusion process, semi-structured observation of the person with a disability, classroom and educational environment, and semi-structured processes of individualized educational intervention planning.
- Observation—the observation process is highly demanding for the teaching staff, but it significantly simplifies all subsequent activities if well-planned and conducted. It benefits from input from all involved with the student and proceeds from the general to the specific observation of spontaneous and facilitated activities through practical tests. Elements of interest during the observation of the students with disabilities include the skills possessed in various areas of development, what they can do consistently, occasionally, and with the teacher’s assistance, how the attitude and approach to interaction (of the student towards the context and vice versa), what the teachers can do to promote their functioning and interaction (what facilitates and what limits), which realistic progress can be most important for the student’s quality of life, and what motivational factors can be used.
- Planning—It refers to identifying the specific educational objectives and strategies to be implemented. Accurate planning cannot be done without comprehensive observation and allows the class staff to know what to do, how, and when. The educational objectives identification should follow the SMART principles (Specific, Measurable, Achievable, Realistic, and Time-bound) [63]. The educational strategies should be selected to pursue the objectives and adapted based on identified facilitators, barriers, and motivational factors. Moreover, the planning process also benefits from discussing with family members and clinicians to align objectives and strategies to pursue a shared goal. Finally, the evaluation time points and the strategies to assess the attainment of the objectives within and at the end of the school year should be planned a priori.

Effectively implementing these elements is mandatory to achieve inclusion.

On the other hand, the benefits that emerged did not last after elementary school ended when the educational approach became more requesting, the social, therapeutic, and motor participation opportunities were reduced, and the age-dependent features of RTT kicked in. From the current results and according to the literature, it is recommended to continuously provide girls and women with RTT of all ages with daily opportunities for social interaction and motor opportunities [57] and physical activity to counteract social withdrawal and motor and musculoskeletal deterioration [58].

### 4.2. Age Groups Comparison

The results from the comparison between the two investigated age groups partially agree with the previous literature. Overall, our sample presents stable clinical and functional characteristics between the two age groups as represented by the total and area scores of the RARS, in line with previous reports [64,65]. In the sample here, the older participants presented the worst scoliosis and feet problems (little, cold feet or presenting with joint abnormalities) reported as stable in adolescents and adults with RTT in a previous report [65]. These discrepancies can be explained by the different age ranges considered to be, in Halbach and colleagues [65], people aged 16 and above were recruited. The current study grouped the participants from the age of 12, as in Cianfaglione et al. [38], and early adolescence and growth spurt are critical years for developing scoliosis in RTT [66,67].

On the other hand, there is initial agreement on the worsening seizure situation in aging people with RTT, in line with our findings [64,65]. Although a previous study reporting people with RTTs’ age-related clinical and behavioral change into adulthood did not find differences between age groups and intensity of repetitive behaviors [38], less frequent bruxism was reported in older people with RTT in our sample. This difference highlights the need for using evaluation tools created explicitly for RTT, such as RARS, allowing assessment of specific RTT characteristics, i.e., Cianfaglione et al. [38] administered the Repetitive Behavior Questionnaire that does not assess for bruxism a repetitive behavior.

The present study presents some limitations, requiring caution in results interpretation. First, specific MeCP2 mutations were available for about half of the participants. The partial comparison between the distribution of the mutations could have biased the comparison as the genotype-phenotype relation was reported in RTT [28,29]. However, the available affected MeCP2 domain distributions were comparable between the two national groups. Moreover, the genotype-phenotype relation should be handled cautiously due to the wide variability of RTT manifestation across genetic mutations [30]. Similarly, the information related to the ongoing rehabilitation treatment was available for 85% of participants. Although no significant difference emerged when comparing the treatment intensities between the two groups, the missing data could limit the validity of this result. Moreover, only two age groups were considered in the current research, as few adults were recruited. A deeper analysis, including more age groups from childhood to adulthood, is needed to better understand the age-related severity of RTT symptoms. Furthermore, no quantitative data on the participants’ socioeconomic status and family composition was available in the investigated datasets. This issue should be explored in future studies. Socioeconomic status and family size and composition may play a significant role in the motor, cognitive, and emotional development of girls with RTT. Due to the absence of these pieces of information in the conducted analysis, the presented results should be interpreted cautiously, and future investigations are required, including a comprehensive analysis of the variables that intervene in the girls’ development.

## 5. Conclusions

The current preliminary study highlighted the differences in functional and clinical characteristics of girls and women with RTT across two countries with different educational care systems. The results support the inclusion of girls with RTT in the mainstream schooling system for a limited daily period, investing in high activity levels within the home and community environments and out of the educational system, and suggest continuously providing the person with RTT with daily occasions of physical activity.

## Figures and Tables

**Table 1:** Number and percentage of subjects carrying a specific MeCP2 mutation. The nine most frequent MeCP2 mutations, according to Bebbington et al. [29], were reported. For each MeCP2 mutation, the corresponding domain category used in the data analysis is indicated. The mutations located neither in the NTD nor MBD were classified as “Others” in line with Good and colleagues [39].

MeCP2 Mutation	MeCP2 Domain	Number of Subjects (%)	
		Israeli	Italian
R168X	Others	5 (14%)	4 (6%)
R255X	Others	3 (9%)	7 (10%)
R270X	Others	4 (11%)	8 (11%)
R133C	MBD	1 (3%)	3 (4%)
T158M	MBD	4 (11%)	13 (18%)
R306C	Others	0 (0%)	7 (10%)
R106W	Others	3 (9%)	1 (1%)
R294X	Others	0 (0%)	4 (6%)
C-terminal deletions	Others	5 (14%)	9 (13%)
Others	Various *	10 (29%)	15 (21%)
Total		35 (100%)	71 (100%)

* Includes: three NTD (two Israeli, one Italian); 10 MBD (two Israeli, eight Italians); and 11 “Others” (six Israeli, six Italians). Abbreviation list: NTD = N-terminal domain; MBD = methyl binding domain.

**Table 2:** Descriptive statistics of RARS areas and total scores for Italian and Israeli age groups and Mann–Whitney U test results.

	U11					U40				
	Italian		Israeli		*p*-Value Ita vs. Is	Italian		Israeli		*p*-Value Ita vs. Is
	Mean (SD)	Median (Range)	Mean (SD)	Median (Range)		Mean (SD)	Median (Range)	Mean (SD)	Median (Range)	
**Cognitive area**	15.7 (4.8)	15.5	15.8 (4.3)	15.5	0.944	15.8 (5.0)	15.0	14.3 (3.4)	14.3	0.190
		(25.0–7.0)		(27.5–7.5)			(26.5–7.5)		(21.0–9.5)	
**Sensory area**	4.0 (1.7)	4.0	3.7 (1.2)	4.0	0.720	4.0 (1.6)	4.0	3.4 (1.4)	3.5	0.101
		(7.0–2.0)		(6.5–2.0)			(8.0–2.0)		(6.5–2.0)	
**Motor** **area**	9.3 (2.8)	9.0	11.6 (3.5)	12.5	0.006 *	9.9 (3.1)	9.5	11.4 (3.3)	11.5	0.020 *
		(16.0–4.0)		(16.0–5.0)			(16.0–5.0)		(16.0–4.0)	
**Emotional area**	7.3 (2.2)	6.5	8.9 (2.4)	9.5	0.014 *	7.4 (1.8)	7.5	7.7 (2.4)	7.0	0.991
		(12.5–4.0)		(12.0–4.0)			(12.0–4.5)		(13.0–4.0)	
**Autonomy area**	11.1 (1.3)	12.0	11.3 (1.2)	12.0	0.406	10.5 (1.8)	11.0	11.2 (1.3)	11.8	0.082
		(12.0–6.0)		(12.0–7.5)			(12.0–5.0)		(12.0–6.0)	
**Disease features**	6.8 (2.1)	6.0	8.5 (2.8)	8.0	0.016 *	7.1 (2.3)	7.0	8.5 (3.1)	8.5	0.013 *
		(12.0–4.0)		(13.0–4.5)			(14.0–4.0)		(15.0–4.0)	
**Behavioral features**	10.2 (2.1)	10.0	11.9 (2.6)	12.0	0.011 *	9.9 (2.1)	10.0	10.8 (3.0)	10.5	0.236
		(15.0–6.0)		(17.0–7.0)			(14.5–6.0)		(18.0–6.0)	
**Total**	67.4 (13.0)	66.5	74.9 (11.7)	75.5	0.039 *	67.6 (12.8)	65.5	70.7 (12.8)	68.0	0.210
		(95.5–37.0)		(95.0–54.0)			(97.0–41.0)		(99.5–46.0)	

*: *p* < 0.05. Abbreviation list: SD = standard deviation; Ita = Italian; Is = Israeli.

## Data Availability

The data presented in this study are available in Supplementary Material.

## Supplementary Materials

The following supporting information can be downloaded at: https://www.mdpi.com/article/10.3390/diagnostics13213390/s1, Table S1: Individual age and RARS items, areas, and total scores for each participant; Table S2a–c: Descriptive statistics and comparison (Mann–Whitney U test) of Italian and Israeli participants all together (not divided for age group—Table S2a) and divided into the two age groups (U11—Table S2b; U40—Table S2c); Table S3: Descriptive statistics and comparison (Mann–Whitney U test) of participants below (U11 group) and above (U40 group) age of 12 years old.
