# Supplementary material for: A Severity Comparison between Italian and Israeli Rett Syndrome Cohorts
Source: Diagnostics (Basel). 2023 Nov 6;13(21):3390. doi: 10.3390/diagnostics13213390 (PMC10648171; doi:10.3390/diagnostics13213390)
Supplement: Supplementary file 1 [file diagnostics-13-03390-s001.zip › diagnostics-2592458-supplementary.pdf]

Table S1: Individual age and RARS items, areas, and total scores for each participant.

Group (1 = IT; 2 = IS)		Age	Cognitive area							Sensory area		Motor area				Emotional area		Autonomy area			Typical features of Rett syndrome													Totals							
			Attention	Spatial orientation	Temporal orientation	Memory	Eye contact, smile response, shared attention	Verbal communication	Non-verbal communication	Sight	hearing	Body	Hands	Scoliosis	Feet	Basic emotion	Emotion of others	Sphincter control	Feeding	Washing and dressing abilities	Mood swings	Convulsions	Crisis of dyspnea	Hyperactivity	Anxiety	Aggressiveness	Bruxism	Oculogyric crises	Epilepsy	Aerophagia	Muscle tension	Food preferences	Overall impression	Cognitive area	Sensory area	Motor area	Emotional area	Autonomy area	Typical pathology features	Typical Behavioral features	Total
IT	1,00	3.00	2.50	4.00	4.00	2.00	1.00	2.50	3.00	2.50	3.00	4.00	3.00	2.50	2.00	1.00	2.00	3.00	4.00	4.00	3.00	1.00	4.00	2.00	3.00	1.00	3.00	1.00	1.50	3.00	2.00	2.50	3.00	19	5.5	11.5	9.00	11	9.50	11.50	80.00
	1,00	3.00	2.00	2.00	2.00	1.00	1.50	2.50	4.00	2.00	2.50	4.00	2.50	1.00	1.50	2.00	2.00	3.00	4.00	4.00	1.50	1.00	2.00	1.50	1.00	1.00	2.00	1.50	1.00	1.50	1.50	1.50	2.50	15	4.5	9	6.50	11	5.50	9.00	63.00
	1,00	3.00	3.00	2.50	2.50	2.00	2.00	3.00	2.00	2.50	2.50	2.00	2.00	2.00	1.00	2.00	2.50	4.00	3.00	4.00	2.50	1.00	1.00	2.00	2.00	1.00	3.00	1.00	1.00	1.00	2.00	1.00	3.00	17	5	7	9.00	11	4.00	10.00	66.00
	1,00	4.00	3.50	4.00	4.00	1.50	3.00	2.50	3.00	2.50	2.50	3.50	2.00	1.00	1.50	1.00	1.50	2.50	3.50	4.00	2.00	1.00	2.00	2.00	2.00	2.00	1.00	1.00	1.00	1.00	2.00	1.00	2.50	21.5	5	8	6.50	10	5.00	9.00	67.50
	1,00	4.00	1.00	1.00	1.50	1.00	1.00	1.00	1.00	1.00	1.00	3.00	2.00	1.00	1.00	1.00	1.00	4.00	4.00	4.00	1.00	1.50	1.00	1.00	1.00	1.00	2.00	2.00	2.00	3.00	1.00	1.00	3.00	7.5	2	7	4.00	12	7.50	8.00	51.00
	1,00	4.00	2.00	2.00	2.00	1.00	1.00	1.00	2.00	1.50	1.00	3.50	1.00	1.00	1.50	1.00	1.50	1.50	4.00	4.00	2.00	2.00	1.50	2.00	1.00	1.50	2.00	1.50	1.50	1.50	1.50	1.00	1.50	11	2.5	7	5.50	9.5	6.50	9.50	53.00
	1,00	4.00	3.00	3.00	2.50	2.50	2.00	4.00	3.50	3.00	2.00	2.00	2.00	2.00	1.00	1.50	1.50	4.00	4.00	4.00	3.00	1.50	1.00	3.50	2.00	1.50	2.00	1.50	1.50	2.00	2.00	3.50	3.50	20.5	5	7	8.00	12	6.00	14.00	76.00
	1,00	5.00	2.50	1.50	2.50	2.50	2.00	2.50	2.50	1.50	1.50	1.50	1.50	1.50	1.00	1.50	2.50	3.50	3.50	4.00	2.00	1.00	1.00	1.50	1.50	2.00	2.00	1.00	1.00	1.00	1.00	1.50	2.50	16	3	5.5	7.50	11	4.00	9.00	58.50
	1,00	5.00	1.00	3.50	2.50	1.00	1.00	3.50	1.50	1.00	1.00	2.00	2.00	1.00	3.00	1.50	2.50	3.00	4.00	4.00	2.00	1.00	3.00	1.00	1.50	1.00	2.00	1.00	1.00	2.50	1.00	1.50	3.00	14	2	8	7.50	11	7.50	7.50	60.50
	1,00	5.00	2.00	1.00	2.00	1.00	1.00	1.00	1.00	1.00	1.00	2.00	2.00	2.00	1.00	1.00	2.00	4.00	4.00	4.00	2.00	1.00	4.00	2.00	1.00	1.00	1.00	1.00	1.00	2.00	2.00	1.00	3.00	9	2	7	6.00	12	8.00	8.00	55.00
	1,00	5.00	2.50	1.50	4.00	1.00	1.50	2.00	1.00	3.00	1.00	3.50	3.00	1.00	2.50	1.00	2.00	4.00	4.00	4.00	2.00	1.00	1.00	1.50	1.00	1.00	2.50	1.50	1.00	1.00	2.00	2.00	3.00	13.5	4	10	6.00	12	4.00	10.50	63.00
	1,00	5.00	3.00	2.00	3.50	2.50	3.00	2.50	3.50	3.00	2.00	4.00	3.00	1.00	1.50	3.00	3.50	4.00	4.00	4.00	3.50	1.00	2.00	2.50	2.00	1.50	3.00	2.00	1.00	2.00	2.50	1.50	4.00	20	5	9.5	12.00	12	6.00	13.00	81.50
	1,00	6.00	3.00	3.00	4.00	2.00	1.50	2.00	2.50	2.50	3.00	4.00	2.50	2.00	3.00	2.00	3.00	4.00	4.00	4.00	2.00	1.00	2.00	2.00	2.00	1.00	2.50	2.50	1.00	2.50	3.00	2.00	3.00	18	5.5	11.5	9.00	12	6.50	13.00	78.50
	1,00	6.00	2.50	1.50	1.50	1.50	1.50	3.00	1.50	1.00	1.50	3.00	3.50	1.50	1.50	2.00	2.00	2.50	4.00	4.00	1.50	1.00	3.00	2.00	1.50	1.00	3.00	1.00	1.00	1.00	2.00	2.00	4.00	13	2.5	9.5	7.00	10.5	6.00	11.00	63.50
	1,00	6.00	3.00	3.50	4.00	1.00	1.50	2.50	4.00	2.50	3.00	2.00	4.00	1.00	1.00	1.00	1.50	3.00	4.00	4.00	2.00	1.00	1.00	2.00	2.00	1.50	3.50	1.50	1.00	2.00	3.00	2.00	3.00	19.5	5.5	8	6.50	11	5.00	13.50	72.00
	1,00	6.00	2.00	2.00	1.50	1.50	1.00	1.50	3.50	1.50	1.00	2.50	2.50	2.50	3.00	1.00	1.50	4.00	4.00	4.00	1.50	1.00	2.00	1.00	1.50	1.00	2.00	2.00	1.00	1.00	2.50	1.00	2.50	13	2.5	10.5	5.50	12	5.00	9.50	60.50
	1,00	6.00	3.00	2.00	4.00	1.50	2.00	2.00	2.50	1.50	1.50	3.50	2.00	2.50	3.00	2.00	2.50	4.00	4.00	4.00	2.00	1.00	1.50	2.00	3.00	1.00	2.00	1.00	1.00	1.00	2.50	1.00	3.00	17	3	11	9.50	12	4.50	9.50	69.50
[illegible]	[illegible]	[illegible]	[illegible]	[illegible]	[illegible]	[illegible]	[illegible]	[illegible]	[illegible]	[illegible]	[illegible]	[illegible]	[illegible]	[illegible]	[illegible]	[illegible]	[illegible]	[illegible]	[illegible]	[illegible]	[illegible]	[illegible]	[illegible]	[illegible]	[illegible]	[illegible]	[illegible]	[illegible]	[illegible]	[illegible]	[illegible]	[illegible]	[illegible]	[illegible]	[illegible]	[illegible]	[illegible]	[illegible]	[illegible]	[illegible]
IS	2,00	21.00	4.00	1.00	1.00	1.00	2.00	3.00	1.00	3.00	1.00	3.00	4.00	4.00	3.50	2.50	2.50	4.00	4.00	4.00	2.00	3.50	2.00	1.00	2.50	1.00	2.00	2.00	1.50	4.00	3.50	3.00	1.50	13	4	14.5	8.00	12	10.50	10.50	76.00
	2,00	21.00	2.00	2.50	1.50	2.00	2.00	4.00	2.50	3.00	2.50	4.00	4.00	4.00	4.00	1.50	2.00	4.00	4.00	4.00	3.00	4.00	4.00	1.00	1.00	1.00	1.50	2.00	4.00	3.00	3.00	2.50	4.00	16.5	5.5	16	7.50	12	15.00	11.00	87.50
	2,00	21.00	3.00	4.00	4.00	1.00	1.50	4.00	1.50	1.50	1.00	3.00	3.00	2.50	2.00	1.00	1.50	4.00	4.00	4.00	2.50	2.50	2.00	1.00	1.50	1.00	2.00	1.50	2.50	1.50	2.50	1.50	3.50	19	2.5	10.5	6.50	12	8.50	9.50	72.00
	2,00	21.00	1.00	3.00	3.50	2.00	1.00	4.00	1.00	1.00	1.00	4.00	4.00	4.00	4.00	1.00	2.00	4.00	4.00	4.00	2.00	4.00	3.00	3.50	4.00	1.00	3.00	2.00	2.00	1.00	4.00	2.00	4.00	15.5	2	16	9.00	12	10.00	15.50	84.00
	2,00	23.00	2.50	1.00	2.00	1.50	2.00	4.00	3.50	2.00	2.00	1.00	3.00	1.00	1.00	1.50	1.50	4.00	4.00	4.00	3.00	1.00	1.00	1.00	1.00	1.00	2.00	1.00	1.00	1.00	1.00	1.50	2.00	16.5	4	6	7.00	12	4.00	7.50	59.00
	2,00	25.00	2.00	2.00	1.00	1.00	1.00	2.00	1.00	1.00	1.00	2.50	2.00	3.50	2.00	1.00	1.00	2.50	4.00	4.00	2.00	2.00	2.50	1.00	1.00	1.00	1.50	1.00	3.00	2.00	2.00	2.00	4.00	10	2	10	5.00	10.5	9.50	8.50	59.50
	2,00	27.00	3.00	3.00	3.00	3.00	2.50	3.50	3.00	2.50	2.50	4.00	2.00	2.00	3.50	3.00	3.00	4.00	4.00	4.00	3.00	1.00	1.00	1.00	3.00	1.50	3.00	3.50	1.00	1.00	3.50	1.50	3.00	21	5	11.5	12.00	12	4.00	14.00	82.50
	2,00	27.00	2.00	2.00	1.50	3.00	2.50	1.50	2.00	3.00	3.00	4.00	2.50	4.00	3.50	1.50	2.00	4.00	4.00	4.00	1.50	2.50	1.50	2.00	3.00	1.00	3.00	1.50	3.50	3.00	3.50	2.00	3.00	14.5	6	14	8.00	12	10.50	13.00	81.00
	2,00	27.00	2.00	1.00	2.00	1.00	1.00	4.00	1.50	1.00	1.00	3.00	1.50	4.00	4.00	1.50	2.00	4.00	4.00	4.00	3.00	2.00	2.00	1.00	1.50	2.00	2.00	1.00	2.00	1.50	3.00	1.50	3.00	12.5	2	12.5	8.00	12	7.50	10.50	68.00
	2,00	28.00	2.00	1.00	1.00	1.00	1.00	2.50	1.00	1.00	1.00	4.00	4.00	2.00	2.00	1.00	1.00	3.00	4.00	4.00	1.00	2.00	3.00	1.00	2.00	1.00	2.00	1.00	4.00	2.00	2.00	1.00	4.00	9.5	2	12	5.00	11	11.00	8.00	62.50
	2,00	31.00	1.50	1.50	2.50	1.00	1.00	4.00	1.50	2.00	1.00	2.00	3.50	4.00	2.00	1.00	2.00	3.50	4.00	4.00	2.00	1.00	1.00	1.00	1.00	1.00	2.50	1.00	1.50	3.00	1.50	1.00	4.00	13	3	11.5	6.00	11.5	6.50	8.00	63.50
	2,00	38.00	4.00	3.00	4.00	1.00	1.50	1.50	2.50	3.00	3.50	2.00	1.00	2.50	1.00	1.00	3.00	3.00	4.00	3.50	4.00	4.00	1.00	1.00	4.00	4.00	4.00	2.50	4.00	4.00	2.00	1.00	4.00	17.5	6.5	6.5	12.00	10.5	13.00	14.50	84.50

Table S2: Descriptive statistics and comparison (Mann-Whitney U test) of Italian and Israeli participants all together (not divided for age group - Table S2a) and divided into the two age groups (U11 - Table S2b; U40 - Table S2c).

Legend: 🔴 : Italian group's score is higher than the Israeli (worst functioning) 🟢 : Italian group's score is lower than the Israeli (better functioning) [shaded, shown in bold]: pval ≤ 0.05

Table S2a.

Desctiptive		Age	Cognitive area							Sensory area		Motor area				Emotional area		Autonomy area			Typical features of Rett syndrome													Totals							
			Attention	Spatial orientation	Temporal orientation	Memory	Eye contact, smile response, shared attention	Verbal communication	Non-verbal communication	Sight	hearing	Body	Hands	Scoliosis	Feet	Basic emotion	Emotion of others	Sphincter control	Feeding	Washing and dressing abilities	Mood swings	Convulsions	Crisis of dyspnea	Hyperactivity	Anxiety	Aggressiveness	Bruxism	Oculogyric crises	Epilepsy	Aerophagia	Muscle tension	Food preferences	Overall impression	Cognitive area	Sensory area	Motor area	Emotional area	Autonomy area	Typical pathology features	Typical Behavioral features	Total
Italian Group All (no. 136)	Mean	🔴 14.54	🔴 2.57	🔴 2.40	🔴 2.61	🟢 1.48	🔴 1.59	🟢 2.71	🔴 2.43	🔴 2.17	🔴 1.79	🟢 2.60	🟢 2.46	🟢 2.31	🟢 2.31	🟢 1.49	🟢 2.09	🟢 3.19	🟢 3.60	🔴 3.95	🟢 2.14	🟢 1.44	🟢 1.86	🔴 1.74	🟢 1.67	🟢 1.29	🟢 2.05	🟢 1.35	🟢 1.56	🟢 2.07	🟢 2.22	🟢 1.39	🟢 2.99	🔴 15.79	🔴 3.96	🟢 9.68	🟢 7.38	🟢 10.75	🟢 6.94	🟢 10.04	🟢 67.53
	SD	7.88	0.77	1.14	1.06	0.60	0.66	1.07	1.17	0.93	0.86	1.10	1.06	0.96	0.92	0.66	0.95	0.99	0.81	0.21	0.61	0.73	0.92	0.68	0.62	0.47	0.77	0.52	0.75	0.90	0.82	0.54	0.70	4.90	1.60	2.97	1.94	1.61	2.22	2.06	12.86
	Median	13.50	2.50	2.00	2.50	1.25	1.50	2.50	2.00	2.00	1.50	3.00	2.25	2.00	2.50	1.00	2.00	3.50	4.00	4.00	2.00	1.00	2.00	2.00	1.50	1.00	2.00	1.00	1.00	2.00	2.00	1.00	3.00	15.00	4.00	9.00	7.00	11.00	6.75	10.00	66.00
	Max	36.00	4.00	4.00	4.00	3.50	3.50	4.00	4.00	4.00	4.00	4.00	4.00	4.00	4.00	3.50	4.00	4.00	4.00	4.00	3.50	4.00	4.00	3.50	3.50	3.00	4.00	3.00	4.00	4.00	4.00	3.50	4.00	26.50	8.00	16.00	12.50	12.00	14.00	15.00	97.00
	Min	3.00	1.00	1.00	1.00	1.00	1.00	1.00	1.00	1.00	1.00	1.00	1.00	0.50	1.00	1.00	1.00	1.00	1.00	2.50	1.00	1.00	0.50	1.00	1.00	1.00	1.00	1.00	0.50	1.00	1.00	1.00	1.00	7.50	2.00	4.00	4.00	5.00	4.00	6.00	37.00
Israeli Group All (no. 59)	Mean	14.39	2.39	2.40	2.19	1.62	1.57	2.97	1.74	1.92	1.63	3.05	2.93	2.85	2.65	1.68	2.19	3.51	3.79	3.92	2.36	2.20	2.01	1.44	1.87	1.36	2.31	1.76	2.11	2.20	2.47	1.86	3.25	14.87	3.54	11.48	8.11	11.22	8.53	11.20	72.21
	SD	7.88	0.83	1.13	0.95	0.74	0.67	1.03	0.90	0.83	0.71	1.05	0.98	1.05	1.20	0.79	1.00	0.86	0.56	0.26	0.82	1.00	0.92	0.78	0.95	0.69	1.01	0.85	1.04	1.18	0.94	0.84	0.67	3.76	1.32	3.36	2.45	1.23	2.97	2.89	12.50
	Median	14.00	2.50	2.50	2.00	1.50	1.50	3.50	1.50	2.00	1.50	3.00	3.00	3.00	3.00	1.50	2.00	4.00	4.00	4.00	2.00	2.00	2.00	1.00	1.50	1.00	2.00	1.50	2.00	2.00	2.50	1.50	3.00	15.00	4.00	11.50	7.50	12.00	8.50	10.50	72.00
	Max	38.00	4.00	4.00	4.00	4.00	4.00	4.00	4.00	4.00	3.50	4.00	4.00	4.00	4.00	4.00	4.00	4.00	4.00	4.00	4.00	4.00	4.00	4.00	4.00	4.00	4.00	3.50	4.00	4.00	4.00	4.00	4.00	27.50	6.50	16.00	13.00	12.00	15.00	18.00	99.50
	Min	3.00	1.00	1.00	1.00	1.00	1.00	1.00	1.00	1.00	1.00	1.00	1.00	1.00	1.00	1.00	1.00	1.00	1.00	2.50	1.00	1.00	1.00	1.00	1.00	1.00	1.00	1.00	1.00	1.00	1.00	1.00	1.00	7.50	2.00	4.00	4.00	6.00	4.00	6.00	46.00
Mann-Whitney U test - All IT vs. All IS		0.953	0.095	0.926	**0.012**	0.304	0.830	0.126	**0.000**	0.082	0.253	**0.007**	**0.004**	**0.002**	**0.035**	0.070	0.505	**0.015**	0.181	0.297	0.143	**0.000**	0.259	**0.000**	0.445	0.941	0.099	**0.001**	**0.000**	0.863	0.069	**0.000**	**0.017**	0.288	0.129	**0.000**	0.071	0.077	**0.001**	**0.015**	**0.025**

Table S2b.

		Age	Attention	Spatial orien	Temporal ori	Memory	Eye contact, s	Verbal communi	Non-verbal comm	Sight	hearing	Body	Hands	Scoliosis	Feet	Basic emotio	Emotion of ot	Sphincter co	Feeding	Washing and	Mood swings	Convulsions	Crisis of dys	Hyperactivity	Anxiety	Aggressiveness	Bruxism	Oculogyric c	Epilepsy	Aerophagia	Muscle tensi	Food preferer	Overall impr	Cognitive ar	Sensory area	Motor area	Emotional ar	Autonomy a	Typical path	Typical Beha	Total
U11 Italian Group (<12yo - no. 55)	Mean	🔴 7.15	🔴 2.55	🟢 2.47	🔴 2.61	🟢 1.51	🟢 1.56	🟢 2.62	🔴 2.41	🔴 2.08	🔴 1.87	🟢 2.80	🟢 2.51	🟢 1.88	🟢 2.11	🟢 1.41	🟢 2.07	🟢 3.35	🟢 3.80	🟢 3.95	🟢 2.14	🟢 1.32	🔴 1.98	🔴 1.75	🟢 1.73	🟢 1.19	🟢 2.19	🟢 1.39	🟢 1.42	🟢 2.05	🟢 2.24	🟢 1.44	🟢 2.99	🟢 15.74	🔴 3.95	🟢 9.30	🟢 7.35	🟢 11.11	🟢 6.76	🟢 10.20	🟢 67.40
	SD	2.24	0.77	1.07	1.02	0.56	0.66	1.08	1.18	0.95	0.90	1.03	0.89	0.87	0.94	0.60	0.95	0.85	0.55	0.20	0.73	0.67	1.02	0.68	0.64	0.34	0.79	0.53	0.67	0.93	0.82	0.59	0.60	4.76	1.68	2.78	2.19	1.31	2.11	2.08	13.03
	Median	7.00	2.50	2.00	2.50	1.50	1.50	2.50	2.00	2.00	1.50	3.00	2.50	2.00	2.00	1.00	2.00	4.00	4.00	4.00	2.00	1.00	2.00	2.00	2.00	1.00	2.00	1.00	1.00	2.00	2.00	1.00	3.00	15.50	4.00	9.00	6.50	12.00	6.00	10.00	66.50
	Max	11.00	4.00	4.00	4.00	3.00	3.50	4.00	4.00	4.00	4.00	4.00	4.00	4.00	4.00	3.00	4.00	4.00	4.00	4.00	3.50	4.00	4.00	3.50	3.50	2.00	4.00	3.00	4.00	4.00	4.00	3.50	4.00	25.00	7.00	16.00	12.50	12.00	12.00	15.00	95.50
	Min	3.00	1.00	1.00	1.00	1.00	1.00	1.00	1.00	1.00	1.00	1.00	1.00	1.00	1.00	1.00	1.00	1.00	1.00	3.00	1.00	1.00	1.00	1.00	1.00	1.00	1.00	1.00	0.50	1.00	1.00	1.00	1.50	7.50	2.00	4.00	4.00	6.00	4.00	6.00	37.00
U11 Israeli Group (≥12yo - no. 21)	Mean	6.24	2.40	2.74	2.21	1.81	1.69	3.07	1.90	1.98	1.74	3.21	3.00	2.90	2.52	1.86	2.62	3.55	3.81	3.98	2.43	2.31	1.88	1.36	1.95	1.45	2.60	1.90	1.93	2.38	2.64	1.95	3.12	15.83	3.71	11.64	8.86	11.33	8.50	11.90	74.90
	SD	2.34	0.83	1.17	1.01	0.84	0.83	0.97	1.16	0.87	0.64	0.97	1.07	1.06	1.26	0.88	1.13	0.89	0.49	0.11	0.83	1.13	0.92	0.67	1.02	0.77	1.07	0.93	1.03	1.35	0.96	0.85	0.79	4.33	1.23	3.46	2.38	1.15	2.82	2.63	11.71
	Median	6.00	2.50	3.00	2.00	1.50	1.50	3.50	1.50	2.00	1.50	4.00	3.50	3.00	3.00	2.00	3.00	4.00	4.00	4.00	2.00	2.00	1.50	1.00	1.50	1.00	3.00	1.50	1.50	2.00	2.50	2.00	3.00	15.50	4.00	12.50	9.50	12.00	8.00	12.00	75.50
	Max	11.00	4.00	4.00	4.00	4.00	4.00	4.00	4.00	4.00	3.00	4.00	4.00	4.00	4.00	4.00	4.00	4.00	4.00	4.00	4.00	4.00	4.00	3.00	3.50	3.50	4.00	3.50	4.00	4.00	4.00	4.00	4.00	27.50	6.50	16.00	12.00	12.00	13.00	17.00	95.00
	Min	3.00	1.00	1.00	1.00	1.00	1.00	1.00	1.00	1.00	1.00	1.00	1.00	1.00	1.00	1.00	1.00	1.00	2.00	3.50	1.00	1.00	1.00	1.00	1.00	1.00	1.00	1.00	1.00	1.00	1.00	1.00	1.00	7.50	2.00	5.00	4.00	7.50	4.50	7.00	54.00
Mann-Whitney U test - U11 IT vs. U11 IS		0.144	0.349	0.354	0.124	0.183	0.633	0.110	0.110	0.642	0.824	0.090	0.054	**0.000**	0.167	**0.017**	0.050	0.159	0.839	0.881	0.245	**0.000**	0.782	**0.007**	0.664	0.266	0.094	**0.032**	**0.033**	0.505	0.088	**0.008**	0.336	0.944	0.720	**0.006**	**0.014**	0.406	**0.016**	**0.011**	**0.039**

Table S2c.

		Age	Attention	Spatial orien	Temporal ori	Memory	Eye contact, s	Verbal communi	Non-verbal comm	Sight	hearing	Body	Hands	Scoliosis	Feet	Basic emotio	Emotion of ot	Sphincter co	Feeding	Washing and	Mood swings	Convulsions	Crisis of dys	Hyperactivity	Anxiety	Aggressiveness	Bruxism	Oculogyric c	Epilepsy	Aerophagia	Muscle tensi	Food preferer	Overall impr	Cognitive ar	Sensory area	Motor area	Emotional ar	Autonomy a	Typical path	Typical Beha	Total
U40 Italian Group (≥12yo - no. 81)	Mean	🔴 19.57	🔴 2.58	🔴 2.36	🔴 2.62	🟢 1.46	🔴 1.60	🟢 2.77	🔴 2.45	🔴 2.22	🔴 1.74	🟢 2.46	🟢 2.43	🟢 2.59	🟢 2.45	🟢 1.54	🔴 2.10	🟢 3.09	🟢 3.46	🔴 3.95	🟢 2.15	🟢 1.53	🟢 1.78	🔴 1.73	🟢 1.62	🔴 1.35	🟢 1.95	🟢 1.33	🟢 1.66	🟢 2.09	🟢 2.20	🟢 1.36	🟢 2.99	🔴 15.83	🔴 3.96	🟢 9.93	🟢 7.41	🟢 10.50	🟢 7.06	🟢 9.93	🟢 67.61
	SD	6.18	0.78	1.19	1.10	0.63	0.66	1.07	1.16	0.92	0.83	1.14	1.16	0.92	0.89	0.70	0.96	1.06	0.93	0.22	0.53	0.76	0.84	0.68	0.61	0.54	0.74	0.52	0.79	0.89	0.82	0.50	0.77	5.03	1.56	3.09	1.78	1.75	2.29	2.05	12.82
	Median	19.00	2.50	2.00	2.00	1.00	1.50	3.00	2.00	2.00	1.50	2.50	2.00	2.50	2.50	1.00	2.00	3.50	4.00	4.00	2.00	1.00	1.50	2.00	1.50	1.00	2.00	1.00	1.50	2.00	2.00	1.00	3.00	15.00	4.00	9.50	7.50	11.00	7.00	10.00	65.50
	Max	36.00	4.00	4.00	4.00	3.50	3.50	4.00	4.00	4.00	4.00	4.00	4.00	4.00	4.00	3.50	4.00	4.00	4.00	4.00	3.50	4.00	4.00	3.50	3.50	3.00	4.00	3.00	4.00	4.00	4.00	3.00	4.00	26.50	8.00	16.00	12.00	12.00	14.00	14.50	97.00
	Min	12.00	1.00	1.00	1.00	1.00	1.00	1.00	1.00	1.00	1.00	1.00	1.00	0.50	1.00	1.00	1.00	1.00	1.00	2.50	1.00	1.00	0.50	1.00	1.00	1.00	1.00	1.00	1.00	1.00	1.00	1.00	1.00	7.50	2.00	5.00	4.50	5.00	4.00	6.00	41.00
U40 Israeli Group (≥12yo - no. 38)	Mean	18.89	2.38	2.21	2.17	1.51	1.50	2.92	1.64	1.88	1.57	2.96	2.89	2.82	2.72	1.58	1.96	3.49	3.78	3.89	2.33	2.14	2.08	1.49	1.83	1.30	2.16	1.68	2.21	2.11	2.38	1.80	3.33	14.34	3.45	11.39	7.70	11.16	8.54	10.82	70.72
	SD	5.98	0.83	1.08	0.93	0.66	0.56	1.08	0.73	0.82	0.75	1.09	0.94	1.06	1.17	0.73	0.85	0.85	0.60	0.31	0.82	0.92	0.93	0.83	0.92	0.64	0.95	0.80	1.05	1.09	0.92	0.83	0.60	3.36	1.37	3.34	2.42	1.27	3.09	2.99	12.82
	Median	17.00	2.00	2.00	2.00	1.00	1.50	3.25	1.50	2.00	1.00	3.00	3.00	2.50	3.00	1.50	2.00	4.00	4.00	4.00	2.00	2.00	2.00	1.00	1.50	1.00	2.00	1.50	2.00	2.00	2.25	1.50	3.00	14.25	3.50	11.50	7.00	11.75	8.50	10.50	68.00
	Max	38.00	4.00	4.00	4.00	3.00	2.50	4.00	3.50	3.00	3.50	4.00	4.00	4.00	4.00	3.50	4.00	4.00	4.00	4.00	4.00	4.00	4.00	4.00	4.00	4.00	4.00	3.50	4.00	4.00	4.00	4.00	4.00	21.00	6.50	16.00	13.00	12.00	15.00	18.00	99.50
	Min	12.00	1.00	1.00	1.00	1.00	1.00	1.00	1.00	1.00	1.00	1.00	1.00	1.00	1.00	1.00	1.00	1.00	1.00	2.50	1.00	1.00	1.00	1.00	1.00	1.00	1.00	1.00	1.00	1.00	1.00	1.00	2.00	9.50	2.00	4.00	4.00	6.00	4.00	6.00	46.00
Mann-Whitney U test - U40 IT vs. U40 IS		0.601	0.165	0.534	0.052	0.723	0.525	0.473	**0.000**	0.062	0.201	**0.026**	**0.033**	0.318	0.139	0.693	0.546	**0.037**	0.070	0.203	0.319	**0.000**	0.093	**0.010**	0.489	0.335	0.321	**0.013**	**0.006**	0.742	0.312	**0.002**	**0.030**	0.190	0.101	**0.020**	0.991	0.082	**0.013**	0.236	0.210

Table S3: Descriptive statistics and comparison (Mann-Whitney U test) of participants below (U11 group) and above (U40 group) age of 12 years old.

Legend: 🔴 :U11 group's score is higher than the U40 (worst functioning) 🟢 :U11 group's score is lower than the U40 (better functioning) [shaded, shown in bold]: pval ≤ 0.05

Desctiptive		Age	Cognitive area							Sensory area		Motor area				Emotional area		Autonomy area			Typical features of Rett syndrome													Totals							
			Attention	Spatial orientation	Temporal orientation	Memory	Eye contact, smile response, shared attention	Verbal communication	Non-verbal communication	Sight	hearing	Body	Hands	Scoliosis	Feet	Basic emotion	Emotion of others	Sphincter control	Feeding	Washing and dressing abilities	Mood swings	Convulsions	Crisis of dyspnea	Hyperactivity	Anxiety	Aggressiveness	Bruxism	Oculogyric crises	Epilepsy	Aerophagia	Muscle tension	Food preferences	Overall impression	Cognitive area	Sensory area	Motor area	Emotional area	Autonomy area	Typical pathology features	Typical Behavioral features	Total
U11 All (<12yo - no. 76)	Mean	🟢 7.49	🟢 2.48	🔴 2.53	🔴 2.52	🔴 1.55	🔴 1.58	🟢 2.69	🔴 2.27	🟢 2.03	🔴 1.82	🔴 2.93	🔴 2.60	🟢 2.19	🟢 2.25	🟢 1.49	🔴 2.19	🔴 3.41	🔴 3.78	🔴 3.94	🟢 2.17	🟢 1.62	🔴 1.96	🟢 1.62	🔴 1.74	🟢 1.24	🔴 2.26	🔴 1.51	🟢 1.60	🔴 2.10	🔴 2.31	🔴 1.56	🟢 3.03	🔴 15.62	🔴 3.85	🟢 9.98	🔴 7.59	🔴 11.13	🟢 7.28	🔴 10.50	🔴 68.99
	SD	2.71	0.77	1.09	1.04	0.64	0.68	1.06	1.21	0.92	0.81	1.04	0.99	1.00	1.06	0.69	1.02	0.86	0.56	0.24	0.75	0.93	0.95	0.68	0.75	0.48	0.86	0.68	0.83	1.02	0.87	0.70	0.71	4.60	1.54	3.17	2.27	1.32	2.42	2.31	12.98
	Median	7.00	2.50	2.50	2.25	1.50	1.50	2.75	2.00	2.00	1.50	3.00	2.50	2.00	2.00	1.00	2.00	4.00	4.00	4.00	2.00	1.00	2.00	1.50	1.50	1.00	2.00	1.00	1.00	2.00	2.00	1.50	3.00	15.50	4.00	9.75	6.75	12.00	7.00	10.00	67.75
	Max	12.00	4.00	4.00	4.00	4.00	4.00	4.00	4.00	4.00	4.00	4.00	4.00	4.00	4.00	4.00	4.00	4.00	4.00	4.00	4.00	4.00	4.00	3.50	3.50	3.50	4.00	3.50	4.00	4.00	4.00	4.00	4.00	27.50	7.00	16.00	12.50	12.00	13.00	17.00	95.50
	Min	3.00	1.00	1.00	1.00	1.00	1.00	1.00	1.00	1.00	1.00	1.00	1.00	1.00	1.00	1.00	1.00	1.00	1.00	2.50	1.00	1.00	1.00	1.00	1.00	1.00	1.00	1.00	0.50	1.00	1.00	1.00	1.00	7.50	2.00	4.00	4.00	6.00	4.00	6.00	37.00

U40 All (≥12yo - no. 119)	**Mean**	19.35	2.52	2.31	2.47	1.47	1.57	2.82	2.19	2.11	1.68	2.62	2.58	2.66	2.54	1.55	2.05	3.21	3.56	3.93	2.21
	**SD**	6.10	0.80	1.16	1.07	0.64	0.63	1.07	1.10	0.90	0.80	1.14	1.11	0.97	0.99	0.71	0.92	1.01	0.85	0.25	0.64
	**Median**	18.00	2.50	2.00	2.00	1.00	1.50	3.00	2.00	2.00	1.50	3.00	2.50	2.50	2.50	1.50	2.00	4.00	4.00	4.00	2.00
	**Max**	38.00	4.00	4.00	4.00	3.50	3.50	4.00	4.00	4.00	4.00	4.00	4.00	4.00	4.00	3.50	4.00	4.00	4.00	4.00	4.00
	**Min**	12.00	1.00	1.00	1.00	1.00	1.00	1.00	1.00	1.00	1.00	1.00	1.00	0.50	1.00	1.00	1.00	1.00	1.00	2.50	1.00
**Mann-Whitney U test - U11 All vs. U40 All**		0.000	0.710	0.137	0.789	0.130	0.996	0.602	0.862	0.619	0.165	0.089	0.644	0.001	0.031	0.809	0.304	0.228	0.050	0.409	0.924

U40 All (≥12yo - no. 119)	**Mean**	1.73	1.88	1.66	1.69	1.34	2.02	1.44	1.84	2.09	2.26	1.50	3.10	15.36	3.80	10.40	7.50	10.71	7.53	10.21	68.61
	**SD**	0.86	0.88	0.74	0.72	0.57	0.82	0.64	0.92	0.95	0.86	0.65	0.73	4.60	1.52	3.23	2.00	1.64	2.65	2.41	12.85
	**Median**	1.50	2.00	1.50	1.50	1.00	2.00	1.00	1.50	2.00	2.00	1.50	3.00	15.00	4.00	10.50	7.00	11.50	7.50	10.00	66.50
	**Max**	4.00	4.00	4.00	4.00	4.00	4.00	3.50	4.00	4.00	4.00	4.00	4.00	26.50	8.00	16.00	13.00	12.00	15.00	18.00	99.50
	**Min**	1.00	0.50	1.00	1.00	1.00	1.00	1.00	1.00	1.00	1.00	1.00	1.00	7.50	2.00	4.00	4.00	5.00	4.00	6.00	41.00
**Mann-Whitney U test - U11 All vs. U40 All**		0.092	0.770	0.951	0.392	0.482	0.027	0.313	0.027	0.961	0.592	0.464	0.352	0.423	0.682	0.378	0.542	0.062	0.533	0.183	0.556
